# Comprehensive Proteomic Analysis Reveals Intermediate Stage of Non-Lesional Psoriatic Skin and Points out the Importance of Proteins Outside this Trend

**DOI:** 10.1038/s41598-019-47774-5

**Published:** 2019-08-06

**Authors:** Edit Szél, Renáta Bozó, Éva Hunyadi-Gulyás, Máté Manczinger, Kornélia Szabó, Lajos Kemény, Zsuzsanna Bata-Csörgő, Gergely Groma

**Affiliations:** 1 Department of Dermatology and Allergology, University of Szeged, Szeged, Hungary; 2 Laboratory of Proteomics Research, Biological Research Centre of the Hungarian Academy of Sciences, Szeged, Hungary; 3 MTA-SZTE Dermatological Research Group, Szeged, Hungary

**Keywords:** Protein-protein interaction networks, Psoriasis

## Abstract

To better understand the pathomechanism of psoriasis, a comparative proteomic analysis was performed with non-lesional and lesional skin from psoriasis patients and skin from healthy individuals. Strikingly, 79.9% of the proteins that were differentially expressed in lesional and healthy skin exhibited expression levels in non-lesional skin that were within twofold of the levels observed in healthy and lesional skin, suggesting that non-lesional skin represents an intermediate stage. Proteins outside this trend were categorized into three groups: I. proteins in non-lesional skin exhibiting expression similar to lesional skin, which might be predisposing factors (i.e., CSE1L, GART, MYO18A and UGDH); II. proteins that were differentially expressed in non-lesional and lesional skin but not in healthy and lesional skin, which might be non-lesional characteristic alteration (i.e., CHCHD6, CHMP5, FLOT2, ITGA7, LEMD2, NOP56, PLVAP and RRAS); and III. proteins with contrasting differential expression in non-lesional and lesional skin compared to healthy skin, which might contribute to maintaining the non-lesional state (i.e., ITGA7, ITGA8, PLVAP, PSAPL1, SMARCA5 and XP32). Finally, proteins differentially expressed in lesions may indicate increased sensitivity to stimuli, peripheral nervous system alterations, furthermore MYBBP1A and PRKDC were identified as potential regulators of key pathomechanisms, including stress and immune response, proliferation and differentiation.

## Introduction

To date, all therapies available for psoriasis only manage symptoms. Understanding alterations that cause the disease is highly important for developing new therapies to better manage the disease.

Our skin connects, and at the same time separates internal the external environment. It is constantly subjected to many different stimuli that requires proper response, through which the skin can influences the function of other organs, like the brain and the endocrine system in a mutual way^1,2^. In psoriasis, the macroscopically healthy looking non-lesional skin harbors alterations that might cause symptoms^3^. One of the most characteristic properties of non-lesional skin is an altered response to mechanical stress or injury^4^ leading to barrier disruption^5^, which leads to an elevated innate immune response^6,7^. Alterations in non-lesional skin are not restricted to keratinocytes. Angiogenesis is also among those mechanisms that is already affected in non-lesional skin, resulting in altered quantity and quality of microvessels^8^. In addition, it is becoming clear that some adaptive immune responses are also altered^9^. Abnormalities in the dermal extracellular matrix composition — such as elevated expression of the oncofetal splice variant of fibronectin^10^, due to altered splicing events^11^ indicate the involvement of dermal fibroblasts^3^. Several matrix metalloproteinases (MMPs), such as MMP-9, previously thought to be increased only in lesions, are now known to be elevated in non-lesional skin compared to healthy skin^12^. There is also evidence for mechanisms in non-lesional skin that contribute to the maintenance of its state. The PRINS long non-coding RNA is induced by stress and nucleic acids, and it is anticipated to have a protective function in psoriasis. PRINS in the non-lesional skin not only decreases inflammatory responses^13^ by inhibiting IL-6 and CCL-5 mRNA translation, but also influences anti-apoptotic mechanisms^14^. Elevated expression of the anti-inflammatory regulator caspase recruitment domain family member 18 (CARD18) in non-lesional skin compared to healthy skin was found to aid the inhibition of inflammatory events^15^. These mechanisms, among many others, highlight the relevance of comparing non-lesional skin to healthy skin.

One of the most effective ways to study different diseases with such a high complexity and to elucidate related mechanisms is to perform a comparative proteomic analysis of protein extracts derived from affected tissues. Previous large-scale treatises including genomic, transcriptomic and proteomic studies have identified psoriasis-related markers playing key roles in the pathomechanism, such as AKR1B10^16^, CSTA^17^, FABP5^18^, PI3^19^, SCCA2^20^, STAT1^16^, STAT3^21^, S100A7^18–20^, S100A8^19,22^ and S100A9^19,20,22,23^, among others, thereby contributing greatly to the better understanding of the disease. However, none of the full scale proteomic studies^17,18,22,24–27^ to the best of our knowledge, compared lesional and non-lesional psoriatic full thickness skin regions, with the inclusion of biopsies from healthy individuals as a reference in the comparison. The inclusion of healthy skin could provide several important additional information. I. Alterations that are similar in non-lesional and lesional skin, but differ from healthy skin, can be detected and used to identify potential novel disease markers or predisposing factors already present in the non-lesional skin. II. The comparison of non-lesional skin to healthy skin might facilitate the identification of inherent characteristics of psoriatic patients that are already present in their healthy-looking skin prior to lesion development. III. Information could be gained about the extent to which the non-lesional skin is affected in respect to lesional alterations. IV. Altered processes in the non-lesional skin that are contrary to the changes of lesional skin could be identified, some of which may contribute to the maintenance of the non-lesional state and serve as novel intervention points for disease management. We aimed to extend previous proteomic studies, in order to get more information regarding the putative alterations mentioned above. Therefore, a complex comparison was performed, where in addition to non-lesional and lesional skin, samples from healthy skin were also included, in a label-free, semi-quantitative proteomic analysis.

## Results

### Proteomic workflow and information on involved donors

**Figure 1:**
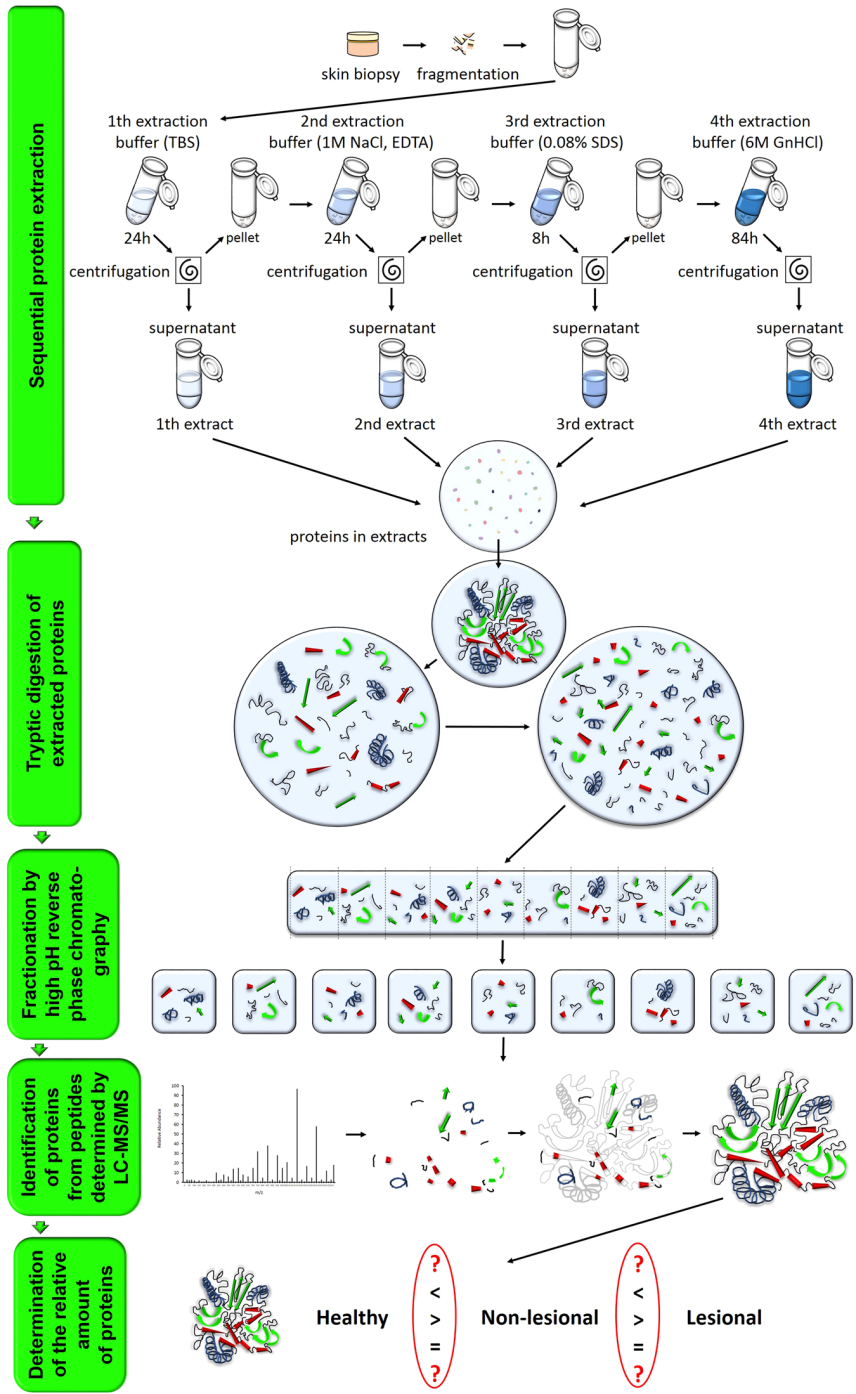
Schematic illustration of the applied proteomic workflow.

Three biological replicas of our proteomic approach were performed following sequential protein extraction of total skin biopsies. Each proteomic replica contained samples from three healthy donors as well as non-lesional and lesional biopsies from three psoriatic patients. The schematic overview of the applied proteomic strategy is summarized in Fig. 1 (also see Supplementary Information: Materials and Methods), and basic demographic and clinical characteristics of psoriatic patients and healthy donors are listed in Table 1. (Criteria for inclusion of patients in the study and skin sample collection are described at Supplementary Material: Materials and Methods section).

**Table 1:** Basic demographic and clinical characteristics of donors involved in the proteomic analysis. (H: healthy donor, P: plaque-type psoriatic patient).

Proteomic experiment	Group of donors	Donors	Age	Gender	PASI score
No. 1.	Healthy	H I.	46	Male	n/a
		H II.	59		
		H III.	51		
	Plaque type psoriasis	P I.	65		17.1
		P II.	63		9.9
		P III.	50		5.5
No. 2.	Healthy	H IV.	23	Female	n/a
		H V.	48		
		H VI.	51		
	Plaque type psoriasis	P IV.	25		9.2
		P V.	62		21.5
		P VI.	70		17.5
No. 3.	Healthy	H VII.	37	Male	n/a
		H VIII.	39		
		H IX.	61		
	Plaque type psoriasis	P VII.	49		22.4
		P VIII.	55		12.1
		P IX.	61		12

### Biological processes associated with differential expression in healthy and lesional skin

**Figure 2:**
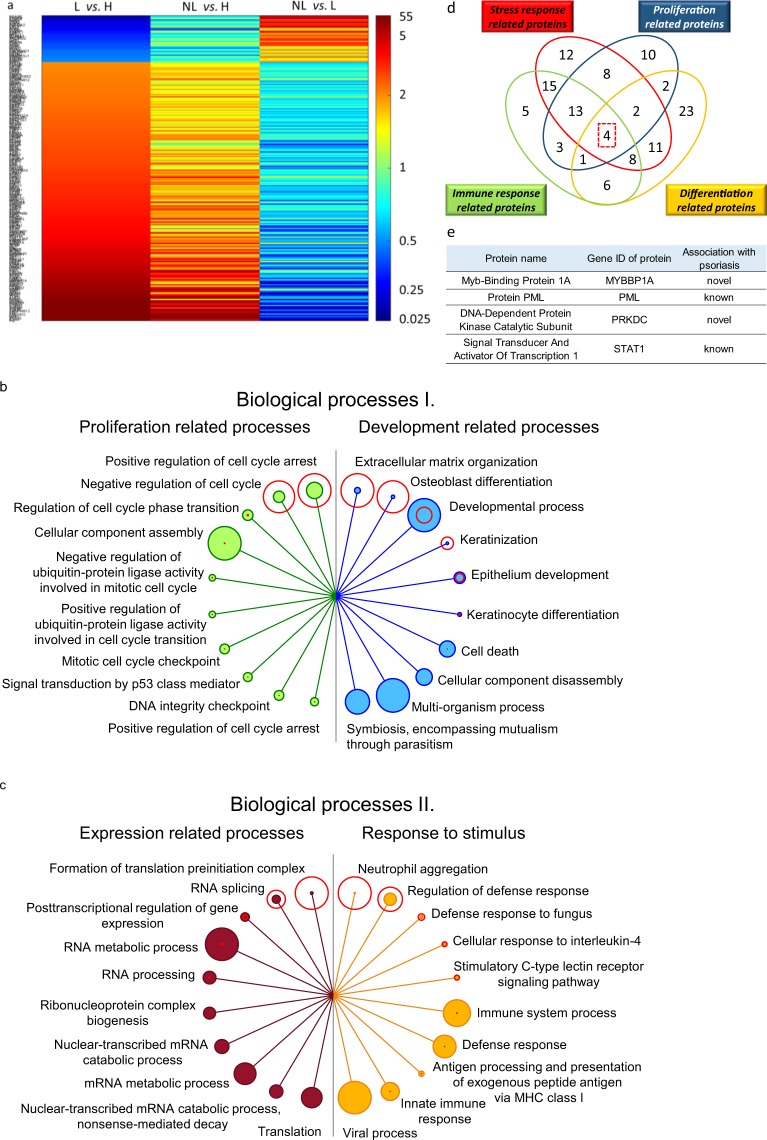
Characterization of altered protein expression of lesional (L) skin compared to healthy (H) skin. Heatmap of relative expression of proteins differentially expressed in L and H skin (**a**, left column), and their expression in non-lesional (NL) and L skin (**a**, middle column) and NL and H skin (a, right column) (**a**). Biological processes for which proteins were differentially expressed in L and H are listed. The top ten processes are depicted for proliferation (**b** left, green circles), development (**b** right, blue circles), expression (**c** left, filled red circles) and response to stimulus (c right, orange circles). False detection rate (FDR) values are indicated with unfilled red circles around the filled circles for the various biological processes. The size of each circle is proportional to FDR values (unfilled circles) or to the number of proteins (filled circles). Four proteins differentially expressed in H and L skin are believed to participate in all four mechanisms of stress, immune response, proliferation and differentiation (**d**) and are listed in (**e**). (*Significant difference in relative protein expression at least by two-fold in L and H comparison).

As an initial step, proteomic results of lesional and healthy skin samples were compared and the relative abundance of 249 proteins was found to be different (Fig. 2a and Supplementary Table 1). A protein–protein interaction-based enrichment analysis was performed with these proteins. We screened for interaction networks and biological processes related to the observed differences in expression using Gene Ontology (GO) analysis of the STRING database (version 10.5). Based on the GO nomenclature and protein composition, the identified biological processes could be classified into the following categories: development, proliferation, regulation of expression and response to stimulus related processes. The ten most significantly different biological processes of each category are listed in Fig. 2b,c and Supplementary Table 2.

Since the major characteristics of psoriatic alterations include altered stress and immune responses as well as dysregulation of proliferation and differentiation, we screened among proteins expressed differentially in lesion compared to healthy skin for central regulators participating in all four of these mechanisms (Fig. 2d). As a result, four central proteins — MYBBP1A, PML, PRKDC and STAT1 — were identified (Fig. 2e).

### Differential protein expression in non-lesional and lesional skin and the biological processes associated with these proteins

**Figure 3:**
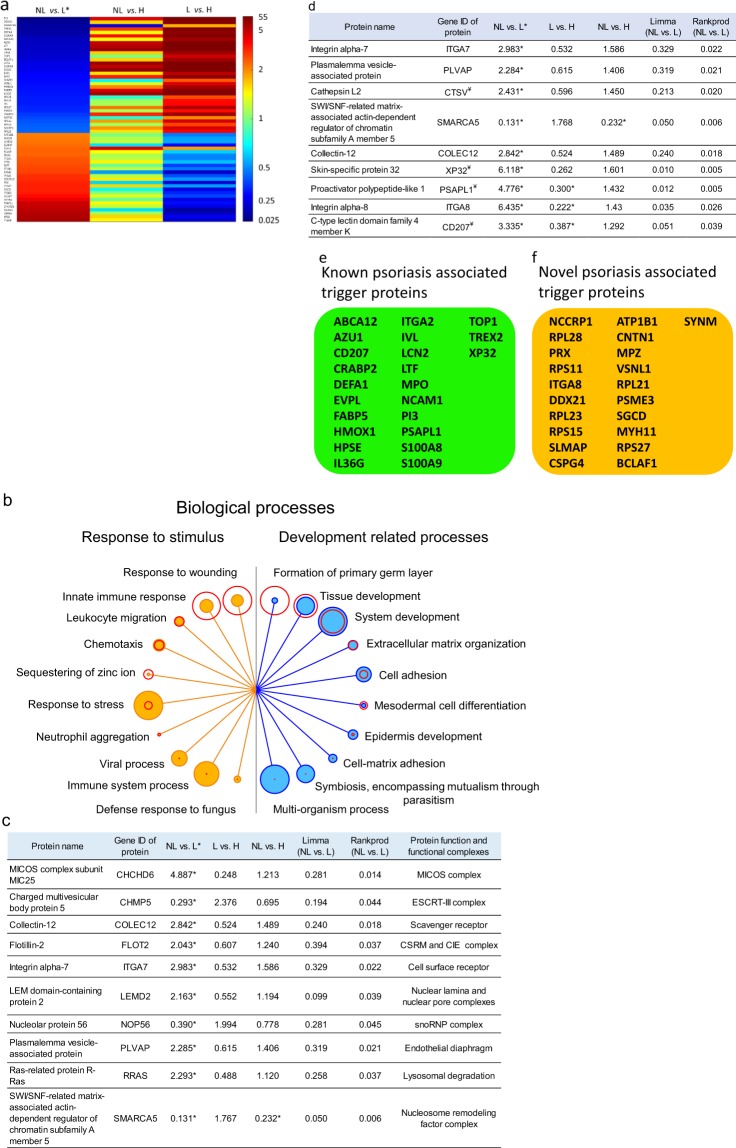
Differential protein expression in lesional (L) and non-lesional (NL) skin and affected biological processes. Heatmap of relative expression for proteins differentially expressed in L and NL skin (**a**, left column) and the relative expression of these proteins NL and L skin (middle column) and L and healthy (H) skin (right column) (**a**). Biological processes for which proteins were differentially expressed in L and NL are listed. The top ten processes depicted to be affected in response to stimulus (**b** left, filled orange circles) and development (b right, filled blue circles). False detection rate (FDR) values are indicated with unfilled red circles around the filled circles for the various biological processes. The size of each circle is proportional to FDR values (unfilled red circles) or to the number of proteins (filled circles) (**b**). Proteins differentially expressed in L and NL but not in H and L are listed (**c**). Proteins for which the changes in NL and L compared to H are in different directions (increased vs. decreased and vice versa) are listed (**d**). Proteins that exhibited altered expression only in lesions (potentially trigger proteins) with known (**e**) and novel (**f**) association with psoriasis are listed. (*Significant difference in relative protein expression at least by two-fold in L and NL comparison).

Comparison of non-lesional and lesional skin proteomes led to the identification of 56 proteins exhibiting at least 2-fold differences in relative abundances. Of these proteins, 32 exhibited higher protein abundance in non-lesional skin compared to lesions, whereas 24 exhibited lower abundance (Fig. 3a and Supplementary Table 3). Functional enrichment analysis of these 56 proteins revealed several biological processes identified in psoriasis pathomechanism, including development, and response to stimulus (Fig. 3b and Supplementary Table 4).

We also found a subset of proteins to be differentially expressed in non-lesional and lesional skin that were not differentially expressed in healthy skin and lesions (Fig. 3c).

The levels of eight proteins were greater in non-lesional skin and lower in lesional skin compared to the levels in healthy skin (non-lesional < healthy < lesional), and one protein exhibited the opposite trend (non-lesional > healthy > lesional). Although the non-lesional and lesional differences in the abundance of these proteins were not statistically significant when compared to healthy skin, the difference in abundance between non-lesional and lesional samples differed significantly by more than two-fold (Fig. 3d).

We also identified 44 proteins that had altered expression only in the comparison of lesional skin to either non-lesional or healthy skin; it is anticipated that these proteins play a role in manifestation and/or maintenance of lesions. The results of a computer-aided, keyword-based literature search suggests that, of these 44 proteins, 23 are already associated with the disease (Fig. 3e), whereas 21 have not yet been associated with psoriasis pathogenesis (Fig. 3f).

Proteins without previous association to psoriasis that exhibited decreased expression in lesions compared to expression in to both non-lesional and healthy skin include modulators of apoptosis, signaling, endothelial cell proliferation, neurite outgrowth, migration, resistance to mechanical stress, cell–cell and extracellular matrix interactions, myelination of peripheral nerves, osmotic and membrane-potential regulation (Supplementary Table 5). In contrast, proteins with increased expression in lesions compared to both non-lesional and healthy skin are involved in cell death, cell proliferation, transcription and translation, calcium sensing (neuronal) and processing of class I MHC peptides (Supplementary Table 5). To further elucidate the significance in psoriasis of the differential expression of these latter three groups of proteins (Fig. 3c,d,f), a detailed automated literature analysis was conducted for associated known functions.

### Comparison of protein expression in non-lesional skin compared to healthy skin

**Figure 4:**
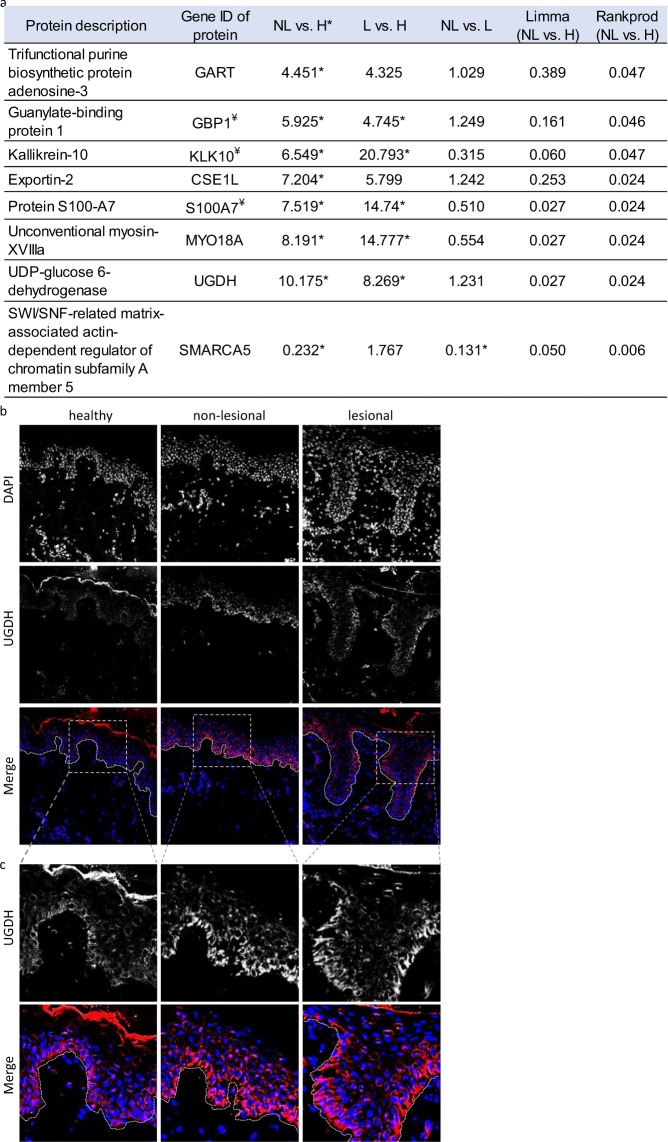
Differentially expressed proteins in non-lesional (NL) and healthy (H) skin. Proteins with expression that differs by at least 2-fold in non-lesional skin and healthy skin are listed (**a**). UGDH protein expression is similarly increased in NL and lesional psoriatic skin, compared to H controls. The highest difference in expression for NL and H was seen with immunohistochemical characterization of UGDH (n = 10), which indicated similar patterns of distribution in the three sample types. The strongest staining was observed in basal keratinocytes, and weaker staining was observed in the upper parts of the epidermis. Higher intensity staining UGDH was observed in non-lesional and lesional skin compared to healthy skin (**b**). A higher magnification of the epidermis is provided (**c**). (In merged figures, DAPI nuclear staining and UGDH are shown in blue and red, respectively; *: indicates statistical significance, ¥: indicates proteins with known association with psoriasis).

Proteins that were differentially expressed in non-lesional skin compared to healthy were also identified. Seven proteins exhibited higher expression levels in non-lesional skin compared to healthy skin and one with lower expression (Fig. 4a). Among these, the relative amount of four proteins (GART, CSE1L, GBP1 and UGDH) was similar in the non-lesional and lesional skin samples. Out of the eight proteins that are differentially expressed in non-lesional skin compared to healthy GBP1, KLK10 and S100A7 have already been associated with psoriasis pathogenesis; the other five are potential novel, early markers of the disease.

To verify our proteomic results, immunofluorescent staining was performed to gain additional information regarding protein localization, deposition and distribution. UGDH had the largest expression differences in non-lesional and healthy skin. As UGDH has not been linked to psoriasis previously, this protein was chosen for further analysis. UGDH staining showed similar epidermal distribution in all three sample types, with the highest protein levels detected in basal keratinocytes (n = 10 different individuals in each group, listed in Supplementary Table 6). Despite the similarities in the UGDH localization pattern, clear differences in staining intensities were observed. The non-lesional and lesional psoriatic samples displayed more robust intensities compared to that of healthy samples, confirming our proteomic results (Fig. 4b,c).

To determine which lesional alterations and to what extent are manifest in non-lesional skin, we selected the 249 proteins that exhibited differential expression in healthy and lesional skin and their expression levels was compared to those in non-lesional skin. In non-lesional skin, the expression of 199 (79.9%) of the proteins differed from the expression in healthy and lesional skin by less than two-fold. Therefore, this category was termed as intermediate, as they may represent a discrete step in the healthy-to-lesional transition (Supplementary Table 7).

### Psoriatic biomarkers, biological functions, canonical pathways and annotation of diseases associated with the detected alterations in protein amounts

To examine the validity of our experimental approach, we further screened our proteomic dataset (lesional vs. healthy) for known, major biomarkers characteristic for psoriasis. These were identified previously in by large scale genomic, transcriptomic and/or proteomic studies. Out of these biomarkers AKR1B10, CSTA, FABP5, PI3, SCCA2, STAT1, STAT3 and members of the S100 family, including S100A7, S100A8, S100A9 were also found in our study. These molecules exhibited elevated expression levels in psoriatic lesions, compared to healthy control skin (Table 2).

**Table 2:** Detected expressional differences of classic protein biomarkers for psoriasis.

Gene ID of protein	L *vs*. H	NL *vs*. H	NL *vs*. L
AKR1B10	32.769*	25.318	0.773
CSTA	2.335*	1.752	0.75
FABP5	15.076*	4.678	0.31*
PI3	52.616*	4.105	0.078
S100A2	5.878*	2.527	0.43
S100A7	14.74*	7.519	0.51
S100A8	20.639*	5.234	0.254*
S100A9	19.679*	3.306	0.168*
SCCA2	35.468*	9.221	0.26*
STAT1	20.504*	14.478	0.706
STAT3	3.766*	2.309	0.613

Further analysis was performed to identify the cellular mechanisms that may be associated with the proteins that were detected in altered amounts in a proteomic approach, using the Ingenuity Pathway Analysis software (IPA). Diseases annotation revealed ‘psoriasis’ as the first hit when lesional and healthy (Table 3), or lesional and non-lesional differences (Table 3) were compared.

**Table 3:** Disease and biological function annotation of differentially expressed proteins.

Categories (L *vs*. H)	Diseases or disease related processes	p-value	Predicted Activation State	Activation z-score	Number of Proteins
**Disease annotation of protein expressional differences between lesional (L) and healthy (H) skin**					
Dermatological Diseases and Conditions, Organismal Injury and Abnormalities	Psoriasis	6.89E-32	—	—	61
	Chronic psoriasis	2.15E-23	—	—	27
Cancer, Cell Death and Survival, Organismal Injury and Abnormalities, Tumor Morphology	Cell death of osteosarcoma cells	4.04E-22	Decreased	−4.899	24
Dermatological Diseases and Conditions, Organismal Injury and Abnormalities	Chronic skin disorder	7.47E-22	—	—	28
Cancer, Cell Death and Survival, Organismal Injury and Abnormalities, Tumor Morphology	Cell death of cancer cells	4.24E-15	Decreased	−4.64	33
Infectious Diseases	Viral Infection	1.89E-12	Increased	3.883	69
	Replication of virus	3.58E-11	—	1.819	34
	Replication of RNA virus	1.93E-10	—	1.799	31
Dermatological Diseases and Conditions, Organismal Injury and Abnormalities	Plaque psoriasis	4.05E-10	—	—	15
Dermatological Diseases and Conditions, Immunological Disease, Inflammatory Disease, Organismal Injury and Abnormalities	Lichen planus	4.86E-10	—	—	13
**Categories (NL vs. L)**	**Diseases or disease related processes**	**p-value**	**Predicted Activation State**	**Activation z-score**	**Number of Proteins**
**Disease annotation of protein expressional differences between non-lesional (NL) and lesional (L) skin**					
Dermatological Diseases and Conditions, Organismal Injury and Abnormalities	Psoriasis	3.72E-15	—	—	21
	Plaque psoriasis	1.78E-11	—	—	10
	Chronic skin disorder	3.06E-10	—	—	10
	Chronic psoriasis	8.58E-10	—	—	9
Immunological Disease	Allergy	8.57E-08	—	—	12
Immunological Disease	Hypersensitive reaction	1.30E-07	—	—	12
Immunological Disease	Immediate hypersensitivity	4.94E-07	—	—	10
Dermatological Diseases and Conditions, Inflammatory Disease, Inflammatory Response, Organismal Injury and Abnormalities	Dermatitis	9.38E-07	—	−1.067	11
Cardiovascular Disease, Organismal Injury and Abnormalities, Renal and Urological Disease	Ischemic acute renal failure	2.99E-06	—	—	3
Organismal Injury and Abnormalities, Reproductive System Disease	Endometriosis	3.13E-06	—	—	10
**Categories (L vs.H)**	**Biological Function**	**p-value**	**Predicted Activation State**	**Activation z-score**	**Number of Proteins**
**Biological function annotation of protein expressional differences between lesional (L) and healthy (H) skin**					
Protein Synthesis	Initiation of translation of protein	3.11E-46	—	—	42
	Translation	3.28E-40	—	0.737	57
	Translation of protein	1.09E-38	—	0.555	55
	Synthesis of protein	3.18E-36	Increased	2.691	64
	Expression of protein	6.76E-36	—	0.527	57
RNA Damage and Repair	Nonsense-mediated mRNA decay	3.06E-35	—	—	32
Protein Synthesis	Metabolism of protein	6.39E-31	Increased	2.92	85
Cell Death and Survival	Necrosis	6.99E-18	Decreased	−2.168	109
RNA Post-Transcriptional Modification	Processing of RNA	4.39E-14	—	−0.577	32
Cellular Movement	Migration of cells	1.1E-12	Increased	2.067	83
**Categories (NL vs. L)**	**Biological Function**	**p-value**	**Predicted Activation State**	**Activation z-score**	**Number of Proteins**
**Biological function annotation of protein expressional differences between non-lesional (NL) and lesional (L) skin**					
Cell Death and Survival	Killing of Staphylococcus aureus	8.53E-10	—	−0.655	5
Cellular Movement,Immune Cell Trafficking	Leukocyte migration	7.62E-08	—	−0.509	18
Cell Death and Survival	Killing of bacteria	1.10E-07	—	−1.608	6
Cell-To-Cell Signaling and Interaction, Reproductive System Development and Function	Binding of gonadal cell lines	1.19E-07	—	1.964	6
Cellular Movement, Hematological System Development and Function, Immune Cell Trafficking	Cell movement of leukocytes	1.55E-07	—	−0.429	16
Cell Death and Survival	Necrosis	2.20E-07	—	−1.927	30
Antimicrobial Response, Inflammatory Response	Antimicrobial response	2.42E-07	Decreased	−2	10
Cell Death and Survival	Killing of Staphylococcus aureus subsp. aureus	5.03E-07	—	—	3
Cellular Movement, Hematological System Development and Function, Immune Cell Trafficking, Inflammatory Response	Cell movement of phagocytes	5.87E-07	—	−0.902	13
Cellular Compromise, Inflammatory Response	Degranulation of cells	6.21E-07	—	−0.87	13

Annotation of biological functions by IPA highlighted ‘initiation of protein translation’ (Table 3) and ‘killing of Staphylococcus aureus’ as the main functions likely to be affected, respectively. Ingenuity canonical pathway screening identified the ‘role of IL-17A in psoriasis’ among the top ten most significant canonical pathways, when either lesional, or non-lesional protein expression was compared to healthy samples (Table 4). In addition, several cancer, neurological and neuromuscular canonical pathways were also highlighted.

**Table 4:** Canonical pathways predicted to be affected in psoriasis based on detected expressional differences of proteins.

Ingenuity Canonical Pathways (L vs. H)	−log (p-value)
EIF2 Signaling	3.69E + 01
Regulation of eIF4 and p70S6K Signaling	1.95E + 01
mTOR Signaling	1.16E + 01
FAT10 Signaling Pathway	4.22E + 00
tRNA Charging	4.01E + 00
Role of IL-17A in Psoriasis	3.32E + 00
RAN Signaling	2.96E + 00
Intrinsic Prothrombin Activation Pathway	2.80E + 00
Polyamine Regulation in Colon Cancer	2.62E + 00
Neuroprotective Role of THOP1 in Alzheimer’s Disease	2.56E + 00
**Ingenuity Canonical Pathways (NL vs. L)**	**−log(p-value)**
Caveolar-mediated Endocytosis Signaling	8.77E + 00
Paxillin Signaling	5.79E + 00
EIF2 Signaling	5.35E + 00
Virus Entry via Endocytic Pathways	4.51E + 00
IL-8 Signaling	4.41E + 00
Integrin Signaling	4.31E + 00
Agrin Interactions at Neuromuscular Junction	4.11E + 00
Regulation of eIF4 and p70S6K Signaling	3.88E + 00
NF-κB Activation by Viruses	3.68E + 00
mTOR Signaling	3.35E + 00
**Ingenuity Canonical Pathways (NL vs. H)**	**−log(p-value)**
UDP-D-xylose and UDP-D-glucuronate Biosynthesis	3.10E + 00
5-aminoimidazole Ribonucleotide Biosynthesis I	2.92E + 00
Tetrahydrofolate Salvage from 5,10-methenyltetrahydrofolate	2.70E + 00
Purine Nucleotides *De Novo* Biosynthesis II	2.36E + 00
Role of IL-17A in Psoriasis	2.29E + 00
Colanic Acid Building Blocks Biosynthesis	2.25E + 00
RAN Signaling	2.17E + 00
Intrinsic Prothrombin Activation Pathway	1.79E + 00
SPINK1 Pancreatic Cancer Pathway	1.66E + 00
MSP-RON Signaling Pathway	1.54E + 00

## Discussion

To expand knowledge about the pathomechanism of psoriasis, many extensive, large-scale comparative proteomic approaches have been performed^17,24,26^. However, the comparison of healthy, non-lesional and lesional skin at the proteomic level has been missing from these studies. To fill this gap, our comparative proteomic analysis included healthy skin as well as non-lesional and lesional psoriatic samples.

In order to check the validity of our proteomic approach we compared major known psoriatic biomarkers published in previous genetic (genome-wide association studies)^21^, transcriptomic^19,21,23^ and proteomic studies^17,18,20,22^ with our proteomic dataset. Known psoriatic lesional biomarkers also found in our study includes AKR1B10^16^, CSTA^17^, FABP5^18^, PI3^19^, SCCA2^20^, STAT1^16^, STAT3^21^, S100A7^18–20^, S100A8^19,22^ and S100A9^19,20,22,23^. Moreover, annotation of diseases resulted in the identification of psoriasis with the strongest correlation based on differentially expressed proteins in either lesional vs. healthy or in lesional vs. non-lesional comparison. Canonical pathway analysis of non-lesional differences compared to healthy skin resulted in the identification of ‘Role of IL-17A in Psoriasis’. However, these annotations also highlighted cancer, neurological, neuromuscular or muscular disease-related mechanism, suggesting their potential involvement in disease pathomechanism, or some similarities between these diseases.

Since our proteomic and *in silico* analysis cannot distinguish between cell-types, and provide information whether mechanistically linked alterations are taking place within the same, or different cell types, further experiments are required in this direction to clarify the exact relevance of these predicted connections to psoriasis pathomechanism.

We performed literature a search for known functions of proteins found to be altered in amounts in our study to suggest mechanism through which they may potentially participate in the pathomechanism of the disease. The detected differences in the expression of proteins in healthy and lesional skin highlighted involvement in psoriasis of cell proliferation^28^, development^29^, response to stimulus^30^, expression^31^ related processes. In the comparison of non-lesional and lesional skin, we identified 56 proteins with differential expression, which represents only 22.5% of the number of proteins which showed altered expression in the comparison of healthy and lesional skin (56 vs. 249). This highlights the importance of studying healthy skin in comparisons using patient samples for pinpointing disease-associated alterations. Qualitative literature-based analysis of these 56 proteins led to the identification of several mechanism for which association with psoriasis has already been described, including processes related to development^29^, response to stimulus^26^ and expression^31^.

Further analysis focused on gaining insight about the extent to which alterations are manifest in lesions and in non-lesional skin. Strikingly, nearly 80% of the 249 proteins exhibiting differential expression in lesional and healthy skin exhibited an intermediate expression level in the non-lesional skin, suggesting the possible presence of early, lesional-like alterations in non-lesional skin. Divergence from this trend was only observed for two small protein groups. Ten proteins — CHCHD6, CHMP5, COLEC12, FLOT2, ITGA7, LEMD2, NOP56, PLVAP, RRAS and SMARCA5 — differed in relative protein amounts in non-lesional and lesional skin, but the amounts of these proteins were similar in healthy and lesional samples. These ten proteins are likely to represent a group of non-lesional characteristic alteration. For nine proteins — CD207, COLEC12, CTSV, ITGA7, ITGA8, PLVAP, PSAPL1, SMARCA5 and XP32 — the direction of the expressional changes was different in non-lesional and lesional samples compared to healthy skin and might represent proteins that contribute to maintaining the non-lesional state.

Next, with the proteins in these two groups, we performed an extensive literature search to suggest potential mechanisms by which they may influence disease pathogenesis. Interestingly, all the identified proteins may play a role in signaling at different levels starting from the cell surface all away to the nucleus or mitochondria. The identified cell surface receptors include two integrins (ITGA7 and ITGA8) that are important in external signal recognition. Decreased ITGA7 levels — as observed in lesional vs. non-lesional skin — could be associated with delayed autophagy^32^, differentiation^33^ and increased migration^34^, all known to be affected in psoriatic lesions. In contrast, elevation of ITGA7 may induce growth suppression^35^. However, ITGA7 is characteristically expressed mainly by smooth muscle cells^36^ in the skin, suggesting that its involvement in keratinocyte related events are at least limited, or none. Instead, may suggest alterations in (vascular) smooth muscle cell adhesion-related processes^37^. Alternatively, ITGA7 may influence neurite outgrowth^38^. Therefore, further studies are required to confirm the observed TGA7 expression alteration and to identify the cell-types of source. Another identified cell-surface molecule, MYO18A, through recognizing microorganism lipopolysaccharides, may increase innate immune responses^39^ promoting cytokine production towards Th1 direction^40^; known to be important in psoriasis^41^.

FLOT2^42^, CHMP5^43^ and COLEC12^44^ participate in endocytic pathways, regulating the levels of cell-surface receptors and, thereby, signaling. FLOT2 is a known component of the raft microdomain complex that represents the major unit regulating STAT signaling pathways according to the raft-STAT signaling hypothesis^45^. The alteration of FLOT2 expression could suggest a high relevance since STAT3 is a key regulator in psoriasis^46^. Reduction of the scavenger receptor COLEC12 could trigger psoriasis by trastuzumab treatment^47^. Moreover, COLEC12 may influence the mitochondrial respiratory chain^48^, and this property is in agreement with the decreased level of mitochondrial MICOS complex subunit CHCHD6^49^ observed in lesions compared to non-lesional skin. CHCHD6 regulates oxygen consumption and thereby may influence cell growth^50^. Reduced levels of CHCHD6 were shown previously to lead to a shift from oxidative metabolism to glycolytic metabolism^51^ that negatively influences keratinocyte differentiation^52^, and both types of mechanisms are known to be affected in psoriasis^53,54^.

The altered expression of LEMD2 may suggest that signal transduction is also altered at the level of the nucleus. LEMD2, located in the inner nuclear membrane, regulates nuclear import/export processes^55^ and thereby intranuclear signaling^50^. During this regulation, LEMD2 is associated with the same complex as CHMP5, which was also identified in our studies. In the nucleus, the STAT-regulated protein NOP56^56^, a core protein of the box C/D small nucleolar ribonucleoprotein (snoRNP) complex, participates in the biogenesis of rRNAs^57^. Increased rRNA biogenesis is suggested to be necessary for high proliferation rate^58^, a process that is crucial for the development of psoriatic lesions.

Abnormal proliferation^28^, differentiation^52^ and, thereby, skin barrier function are key processes during psoriatic plaque formation. SMARCA5 is a component of the nucleosome remodeling factor complex^59^. Decreasing SMARCA5 levels are required for basal keratinocytes to shift form proliferation toward differentiation^60^. XP32 is also a component of the epidermal differentiation complex^61^ and associated with skin barrier function^62^. The observed contrasting expressional differences of these two proteins in the non-lesional and lesional skin may contribute to our understanding of lesion formation and how non-lesional skin maintains its state.

Overall, our results indicate that dysregulation of cellular signaling — from signal detection, through endocytosis of receptors and transduction of signal from the cell surface to the nucleus — may be affected during the disease. The alteration of these systems is likely to lead to increased reaction to external signals that could contribute to the maintenance of psoriatic plaques.

By comparing non-lesional and healthy skin, differential expression was observed for eight proteins (CSE1L, GART, GBP1, KLK10, MYO18A, S1007A, SMARCA5 and UGDH). Four of these proteins (CSE1L, GART, GBP1, UGDH) might be predisposing factors, as their expression was similar in non-lesional and lesional skin, and their significance would have been missed in comparisons for which healthy samples were not included. Of these, UGDH was detected with the highest relative difference. UGDH has not been highlighted previously in association with psoriasis. We therefore decided to analyze it further. Immunohistochemical analysis confirmed our proteomic results: higher UGDH levels were found in non-lesional and lesional skin compared to healthy skin, that was mainly associated with keratinocytes. Elevated UGDH levels may increase chondrocytes proliferation indirectly, likely through increased hyaluronan production that binds different cytokines^63^. However, *in vitro* downregulation of UGDH and consequently decreased hyaluronan amounts did not influence keratinocyte proliferation^64^. These results are in line with our observation, suggesting that elevated UGDH levels observed in non-lesional keratinocytes are not sufficient to modify their proliferation.

Proteins for which expression was affected only in lesions are often considered “trigger” proteins, as changes in the expression of these proteins are linked to the shift of the disease state. The proteins that have not previously been associated with psoriasis were categorized into two groups. The first group of proteins might contribute to the mechanosensitivity of the tissue (SGCD^65^, SYNM^66^, MYH11^67^, ATP1B1^68^). The second group functions within the nervous system (MPZ, PRX^69^, CSPG4^70^, CNTN1 and ITGA8^71^, ATP1B1^72^), could suggest the involvement of the peripheral nerve system in psoriasis^73^.

Finally, we searched for potentially central proteins in disease pathogenesis participating in key mechanisms of psoriasis including regulation of stress and immune response, proliferation and differentiation. Some of the identified proteins, such as PML^74^ and STAT1^75^, have already been linked to psoriasis. We also identified two proteins, PRKDC and MYBBP1A, which have not previously been highlighted in context with the disease. The PRKDC may plays a role in the detection and repair of breaks in double-stranded DNA^76^ and mediates the phosphorylation of c-MYC^77^ and p53^78^ suggesting a potentially important role in psoriasis. The suggested altered expression by our results of the transcription factor MYBBP1A may also be among the potentially important proteins in psoriasis implicated in the pathogenesis since it functions as a co-repressor of NF-κB that may regulate responses to stress and cytokines^79^.

Taken together, our comparative proteomic approach of healthy, non-lesional and lesional skin led to the identification of various proteins which may function in psoriasis pathogenesis, providing a strong base for future studies. Proteins exhibiting opposite expression changes in lesional and non-lesional samples compared to healthy skin may function in the maintenance of the non-lesional stage and may represent future targets for therapeutic purposes.

## Materials and Methods

### Ethics

Skin biopsy collection from donors, the procedure of collection and all experimental protocols were approved by the Regional and Institutional Research Ethics Committee and by the Human Investigation Review Board of the University of Szeged (SOEDAFN-002, IF-562-5/2016 and IF-15056/2015; 157/2015; 3638 and 2799, 3517), strictly following the guidelines and regulations of the Declaration of Helsinki. Prior to surgical intervention and following a detailed description of the skin biopsy donation procedure, participants provided written informed consent. No donor under the age of 18 was included in our study.

### Criteria for inclusion of patients in the study and skin sample collection

To identify alterations that are general in chronic plaque psoriasis and keep the number of volunteers for skin biopsy collection to a minimum, for our proteomic approach, we randomly engaged individuals (I) of different age to minimize possible age-related differences; (II) with various Psoriasis Area Severity Index (PASI) scores between 5 and 25, since the score for an individual patient varies over time and with relapse; (III) of both genders to avoid possible gender-associated differences; and (IV) with both early and late onset. A total of 9 (3 × 3) patients suffering from chronic plaque psoriasis and the same number of healthy donors were involved in our study. The data of individuals involved in the study are summarized in Table 1. All psoriatic patients had not received any kind of treatment for the condition for at least 6 months. The 6 mm skin punch biopsies containing the epidermis and the dermis were collected from an area of the upper-middle gluteal region that is not exposed to sunlight. Both lesional and non-lesional samples were collected from patients. Non-lesional samples were taken at least 7 cm from the edge of the lesion subjected for biopsy. The presence of psoriasis was clinically verified for all patients, and clinical as well as demographic data of the donors are presented in Table 1.

### Comprehensive and comparative proteomics of healthy, non-lesional and lesional skin

#### Sample preparation from skin biopsy and sequential protein extraction

Samples were cut with a razor blade. Skin proteins were extracted sequentially in four consecutive solubility-based extraction steps. Extraction buffers were used in increasing order of their solubilizing properties for a better separation of proteins. Samples were initially incubated in extraction buffer I. (0.15 M NaCl, 50 mM Tris-HCl, pH 7.4) for 24 h at 4 °C in the presence of protease inhibitors. Protein extracts were then clarified by centrifugation and separated from the pellet. This step was repeated by resuspending the pellet in extraction buffer II, which contained 1 M NaCl, 25 mM EDTA, 50 mM Tris-HCl, pH 7.4. Following extraction with 250 mM SDS-containing extraction buffer III (8 h at room temperature), guanidine hydrochloride containing extraction buffer IV (4 M GuHCl, 10 mM EDTA, 50 mM Tris-HCl, pH 7.4) was applied for 48 h at 4 °C. The same protein extracts of the three donors were pooled in each investigated group (healthy, non-lesional, lesional). Extraction procedure was carried out three times and each contained extracted proteins of three donors following the pooling of the samples which were than subjected for downstream proteomic analysis.

#### Protein identification by 2D LC-MSMS

A total 35 µg protein from each sample was applied for mass spectrometry analysis. A modified filter-aided sample preparation method was used for tryptic digestion of the protein extracts^80^. High-pH reversed-phase chromatography was performed on a C18 column (Phenomenex, Kinetex 5 µ EVO C18 100 A, 2.1 × 100 mm; cat. no. 00D-4622-AN, flow rate: 150 µl/min). Forty-eight fractions were collected from 1 to 25 minutes (half minute/fraction) and 4-4 fractions were combined (1,13,24,37; 2,14,25,38 and so on) to get 12 final fractions. Each fraction was subjected to nano LC-MSMS analysis on an Orbitrap Elite hybrid mass spectrometer (Thermo) coupled with a Waters nanoAcquity UPLC system, using a gradient elution after trapping the samples onto the trap column. Data-dependent analyses were applied; the 20 most intense peaks were selected for ion-trap collision-induced dissociation after each survey scan measured in the Orbitrap. Proteome Discoverer (ver.: 1.3) was used to generate MS/MS peak-list files and our in-cloud ProteinProspector (ver.: 5.16.0) database search engine was used for protein identification against the human sequences from the UniProtKB.2015.12.14.random.concat (149781/55820795 entries searched) database. Detailed protocols and applied counting for semiquantitative analysis is described as Supplementary Information.

### Immunofluorescence staining of skin sections for UGDH

For immunofluorescence analysis, 5 µm sections of frozen embedded skin biopsies from psoriatic patients (non-lesional and lesional skin) and healthy individuals were used. After fixation and permeabilization (Foxp3 staining buffer set, fixation/permeabilization kit, Miltenyi Biotec, used according to the description of the manufacturer), samples were blocked in Tris-buffered saline (TBS) containing 1% bovine serum albumin (BSA) and 1% normal goat serum (NGS) for 1 h at room temperature. Samples were incubated overnight at 4 °C in TBS with 1% NGS and primary antibodies against UGDH (rabbit polyclonal antibody, ab155005, Abcam), diluted to 1:100. Following washing in TBS, AF546 secondary antibodies (Goat anti-Rabbit IgG (H + L) Highly Cross-Adsorbed Secondary Antibody, Alexa Fluor 546, A-11035, Invitrogen), diluted in TBS containing 1% NGS to 1:500, were applied for 1 h at room temperature.

### Literature search to identify novel psoriasis-associated proteins

To identify proteins not yet linked with the pathomechanism of psoriasis, literature mining was carried out using protein names or the encoding gene’s HUGO Gene Nomenclature Committee (HGNC) symbol(s), applying the following strategy: each protein or gene name was searched together with “psoriasis” as a keyword using the RISmed R package.

### Statistical analysis

To compare protein abundance from healthy, lesional and non-lesional skin extracts, significant differences were determined based on relative peptide ion chromatograms and spectrum counting and evaluated using two different approaches: (1) modified t-test (limma) and (2) rank product test (as described by Schwämmle *et al*.^81^) following t-test. We considered a protein amount to be different between two samples if at least one of the three tests were significant (test <0.05) and the absolute fold change was at least two or higher.

## Supplementary information

Supplementary information
