# Supplementary material for: Comprehensive Proteomic Analysis Reveals Intermediate Stage of Non-Lesional Psoriatic Skin and Points out the Importance of Proteins Outside this Trend
Source: Sci Rep. 2019 Aug 6;9:11382. doi: 10.1038/s41598-019-47774-5 (PMC6684579; doi:10.1038/s41598-019-47774-5)
Supplement: Supplementary file 1 — Supplementary information [file 41598_2019_47774_MOESM1_ESM.docx]

comprehensive proteomic analysis reveals Intermediate staGe of non-lesional psoriatic skin and points out the importance of PROTEINS OUTSIDE this trend

Supplementary Information

Edit Szél^1^, Renáta Bozó^1^, Eva Hunyadi-Gulyas^2^, Máté Manczinger^1,3^, Kornélia Szabó ^1,3^, Lajos Kemény^1,3^, Zsuzsanna Bata-Csörgő^1,3^, Gergely Groma^1,3^

^1^Department of Dermatology and Allergology, University of Szeged, Hungary; ^2^Laboratory of Proteomics Research, Biological Research Centre of the Hungarian Academy of Sciences, Szeged, Hungary; ^3^MTA-SZTE Dermatological Research Group, Szeged, Hungary

Correspondence: Gergely Groma, PhD

e-mail: [groma.gergely@med.u-szeged.hu](mailto:groma.gergely@med.u-szeged.hu)

**1.** **Supplementary Materials and Methods**

**Sample preparation for 2D LC-MSMS analysis and protein digestion**

A total 35 µg protein from each sample was used for mass spectrometry analysis. A filter-aided sample preparation (FASP) method was used for digestion, according to the method of Wiśniewski *et al.* The following modified protocol was used, depending on the extraction buffers. The pH of all samples was adjusted to >pH7 with 1.5 M ammonium bicarbonate buffer (ABC). Guanidine hydrochloride (GuHCl) was added to extracts which did not contain SDS to denature the proteins. After reduction with dithiothreitol (DTT) and alkylation with iodoacetamide (IAM) steps, samples were transferred to a 30 kDa MWCO filter tube (Millipore), the buffer was exchanged with 25 mM ABC and the samples were subjected to digestion using trypsin (Promega, side change protected enzyme). After overnight digestion, the peptides were collected by centrifugation, dried in rotatory vacuum centrifuge and resuspended in 10 mM ABC (pH 10) prior to high-pH fractionation.

**2D LC-MSMS analysis**

High-pH reversed-phase chromatography was performed using an Eldex micro HPLC pump (SunChrom, Germany) on a reversed phase column (Phenomenex, Kinetex 5 µ EVO C18 100 A, 2.1 x 100 mm; cat. no. 00D-4622-AN). The elution flow rate was 150 µl/min using the following gradient: 5–40% B for 10 minutes, 40–95% B for 2 minutes, 95% B for 3 minutes, 95–5% for 2 minutes and 5% B for 7 minutes (mobile phase A was 10 mM ABC, pH 10, and mobile phase B was the mixture of 10% A and 90% acetonitrile). Forty-eight fractions were collected from 1 to 25 minutes (half minute/fraction) and 4-4 fractions were combined (1,13,24,37; 2,14,25,38 and so on) to get 12 final fractions. Each fraction was dried in a vacuum centrifuge, resolved in 0.1% FA in water and subjected to nano LC-MSMS analysis on an Orbitrap Elite hybrid mass spectrometer (Thermo). Nano UPLC runs were performed on a Waters nanoAcquity UPLC system, using a gradient elution after trapping the samples onto the trap column (186007238 Waters Symmetry C18, 0.180 mm ×20 mm, 5 µm, 100 Å) with 3% B (mobile phase B: 0.1% formic acid in acetonitrile, mobile phase A: 0.1% formic acid in water) for 2 minutes with a flow rate of 10 µl/min. The analytical separation was performed with the following gradient elution: 3–10% B for 5 minutes and to 40% for 32 minutes, followed by a double wash at the end of the gradient to reduce carry-over to the next run. The flow rate was 200 nl/min; the column (186003545 Waters BEH130 C18, 0.075 mm×250 mm, 1.7 µm, 130 Å) was regulated with a thermostat to 60°C. Data-dependent analyses were applied; the 20 most intense peaks were selected for ion-trap collision-induced dissociation (CID) after each survey scan. The survey spectra were measured in the Orbitrap (mass range: 380–1400 m/z; resolution: 120’000 @ 400 m/z), while the CID MS2 spectra were detected in the ion trap. Selected precursor masses were dynamically excluded for 30 seconds to facilitate more comprehensive analysis of the samples.

**Data evaluation**

Proteome Discoverer (ver.: 1.3) was used to generate MS/MS peak-list files and our in-cloud ProteinProspector (ver.: 5.16.0) database search engine was used for protein identification. The twelve peak-list files related to one sample were merged and subjected to database search using human sequences from the UniProtKB.2015.12.14.random.concat (149781/55820795 entries searched) database. Only fully specific tryptic peptides with a maximum of 2 missed cleavage sites were considered. Variable modifications were set to the following: carbamidomethyl Cys as constant, Met and Pro oxidation, peptide N terminal pyroglutamine formation from Gln, Asn and Gln deamidation and protein N terminal acetylation. Error tolerance for precursor ion was set to 5 ppm and 0.6 Da for fragment masses. Maximum of 5 modifications per peptides were allowed.

*Semiquantitative evaluation by spectral counting*

The calculated false discovery rates (FDR) were less than 1% in all cases; two further criteria were necessary to consider a protein hit as real. At least in one of the groups of the three different skin extract types (healthy, non-lesional, lesional) must be identified the protein from all three replicates and at least 3 unique peptides match the protein in all 3 replicates. The base of our semiquantitative analysis is the number of spectra that identifies the particular protein (peptide count) divided by the total number of peptide spectrum match (PSM) of the sample. To avoid the calculation problem presented when a protein has a spectral count of zero in one of the samples, the following formula were used to calculate the relative spectral counts: $RPC=\frac{n+f}{t+f}$, where RPC is the relative peptide count, *n* is the number of spectra identified the protein, *t* is the total number of identified spectra in the sample, *f* is a correction factor, which is set to 1. A t-test (two sample t-test, assuming unequal variances) was performed to decide whether the difference between the two groups (with 3 replicates) was significant. The averages of the relative peptide counts were compared, and at least two-fold changes were considered as real. It was assumed that most of the proteins do not show quantitative changes, and the relative peptide count ratio normalization by the median value can be performed.

**2. Supplementary Tables and Figures**

Supplementary Table 1. Relative expression ratios of proteins detected in different amounts in lesional (L) skin compared to healthy (H), and their expression ratios in non-lesional (NL) vs. H and NL vs. H comparisons.

Supplement Table 2.

Supplementary Table 3. Relative expression ratios of proteins detected in different amounts in non-lesional (NL) skin compared to lesional (L), and their expression ratios in lesional (L) vs. healthy (L vs. H) and non-lesional vs. healthy (NL vs. H) comparisons.

Supplementary Table 4.

Supplementary Table 5.

Supplementary Table 6.

Supplementary Table 7.
